# Rapid Bacteria Detection from Patients’ Blood Bypassing Classical Bacterial Culturing

**DOI:** 10.3390/bios12110994

**Published:** 2022-11-09

**Authors:** François Huber, Hans Peter Lang, Stefanie Heller, Julia Anna Bielicki, Christoph Gerber, Ernst Meyer, Adrian Egli

**Affiliations:** 1 Swiss Nanoscience Institute (SNI), Department of Physics, University of Basel, CH-4056 Basel, Switzerland; 2 Applied Microbiology Research (Lab 315), Zentrum für Lehre und Forschung, Department of Biomedicine, University of Basel, CH-4031 Basel, Switzerland; 3 University Children’s Hospital Basel (UKBB), Department of Medicine, University of Basel, CH-4056 Basel, Switzerland; 4 Clinical Bacteriology and Mycology, University Hospital Basel, CH-4031 Basel, Switzerland; 5 Institute of Medical Microbiology, University of Zurich, CH-8006 Zurich, Switzerland

**Keywords:** bacteremia, sepsis, nanomechanical biosensors, cantilevers, bacterial infections, total RNA, blood samples, rapid sensitive diagnostics

## Abstract

Sepsis is a life-threatening condition mostly caused by a bacterial infection resulting in inflammatory reaction and organ dysfunction if not treated effectively. Rapid identification of the causing bacterial pathogen already in the early stage of bacteremia is therefore vital. Current technologies still rely on time-consuming procedures including bacterial culturing up to 72 h. Our approach is based on ultra-rapid and highly sensitive nanomechanical sensor arrays. In measurements we observe two clearly distinguishable distributions consisting of samples with bacteria and without bacteria respectively. Compressive surface stress indicates the presence of bacteria. For this proof-of-concept, we extracted total RNA from EDTA whole blood samples from patients with blood-culture-confirmed bacteremia, which is the reference standard in diagnostics. We determined the presence or absence of bacterial RNA in the sample through 16S-rRNA hybridization and species-specific probes using nanomechanical sensor arrays. Via both probes, we identified two clinically highly-relevant bacterial species i.e., *Escherichia coli* and *Staphylococcus aureus* down to an equivalent of 20 CFU per milliliter EDTA whole blood. The dynamic range of three orders of magnitude covers most clinical cases. We correctly identified all patient samples regarding the presence or absence of bacteria. We envision our technology as an important contribution to early and sensitive sepsis diagnosis directly from blood without requirement for cultivation. This would be a game changer in diagnostics, as no commercial PCR or POCT device currently exists who can do this.

## 1. Introduction

A recent study by K. E. Rudd et al. [1] estimated that in 2017 there were 48.9 million cases of sepsis and 11 million sepsis-related deaths worldwide. Furthermore, children account for almost half of all global sepsis cases, with an estimated 20 million cases and 2.9 million global deaths in children under the age of five years. In clinical management of a septic patient, the rapid and reliable identification of the causing bacterial pathogen is of high importance [2]. Especially blood culture-based diagnosis is a reference standard as it allows for identification of blood stream infection and adaptation of empiric treatment based on the bacterial species. The usage of blood culture bottles has a series of downfalls during the diagnostic process. First of all, the sensitivity of blood cultures during sepsis is about 67.7% [3] and requires a long time from collection to identification of a pathogen. The blood culture is incubated for up to 48 h until it turns positive, then a subculture is performed in order to obtain single bacterial colonies. Finally, matrix-assisted laser-desorption/ionization time of flight (MALDI-TOF) mass spectrometry is used for species identification. This culture-based identification workflow requires up to 72 h. Molecular diagnostic testing such as polymerase chain reaction (PCR), isothermal amplification [4] or panel/solid phase PCRs [5] may overcome some of these obstacles by allowing identification directly from positive blood culture. Automated and semiautomated devices, such as: Becton Dickinson Phoenix, Siemens MicroScan Walk Away or Vitek 2, have been extensively used in clinical settings [6], mostly for fast growing bacteria. Recently machine learning algorithms were introduced to identify bacteria using mass spectrometry data [7]. But all these methods are time consuming and require expensive equipment as well as bulky infrastructure. But, these technologies may also be challenging due to inhibitory substances and sensitivity issues [8,9], especially when applied directly on blood [10]. Ubiquitously available point of care testing (POCT) devices based on paper strips, arrays, beads and microfluidics are easy to use, fast, but less reliable and less sensitive [11].

A relatively new method [12,13] based on atomic force microscopy (AFM) measures the vibrational motion of bacteria in real time. Nevertheless, single bacterial colonies from previous cultures are still needed. After preselection on specific culture media the method can be applied to slow growing bacteria as well, but still requiring up to 12 h to provide reliable results.

We recently introduced a method based on a cantilever nanosensor diagnostic assay for direct detection of bacteria (see Material and Methods) that may overcome some of the above-mentioned problems [14]. The method provides a fundamentally different approach, with internal references for differential readout to eliminate thermal drift and nonspecific binding artifacts. Additionally, inhibitory substances pose no challenge to nanosensor-based assays, since no enzymatic reactions are required. Such a setup utilizes ultra-sensitive sensors for the detection of biochemical interactions in liquid environments [15,16]. We have previously shown that nanosensors are highly specific, sensitive and fast tools for the detection of bacterial antibiotic resistance genes. The technology is able to detect 10 fg/µL total RNA corresponding to less than 10 bacterial cells at single nucleotide polymorphism (SNP) specificity in less than 5 min from cultured bacteria [17]. The sensitivity is high enough to detect pathogens already in the early stages of bacteremia.

We now show that this assay is able to conclusively detect bacteria directly using RNA extracted from EDTA whole blood samples of patients with positive blood cultures (reference standard), thereby bypassing time-consuming bacteria culturing as well as amplification or labelling. Each nanosensor is coated with 16S-rRNA specific and species-specific targeted oligonucleotides for molecular recognition. Hybridization of the bacterial RNA sequence is mechanically transduced to the cantilever surface, resulting in bending of the cantilever due to compressive stress. In this proof-of-concept study, we first focus on the detection of bacteria in general, in bacteremia, and furthermore on the specific detection of two most relevant bacterial species using species-specific probes for *Staphylococcus aureus* and *Escherichia coli* which account for more than 50% of sepsis cases. Focus is on proof-of-concept for a novel technique for rapid and sensitive detection of bacteremia directly from EDTA whole blood of patients using a compact device.

## 2. Materials and Methods

### 2.1. RNA Preparation from Blood

To determine the level of rejection (LOR), the bacteria (*Escherichia coli* ATCC 25922 and *Staphylococcus aureus* ATCC 29213) were grown to OD_600nm_: 0.5 in Mueller Hinton broth. A 1:10 serial dilution was performed to obtain bacteria concentration from 10^8^ CFU/mL to 0 CFU/mL. The concentrations were confirmed by classical culture and manual counting of the CFUs along the concentration gradient. Next, the different bacteria concentrations were mixed with EDTA whole blood in a 1:1 ratio.

For performance assessment of clinical samples, we collected EDTA whole blood samples from patients who showed a positive blood culture with either *Escherichia coli* or *Staphylococcus aureus* at the University Hospital Basel. The bacteria identification of the blood culture positive material was done with a MALDI-TOF mass-spectrometry system (Bruker). We also included culture-negative EDTA whole blood samples as negative controls.

For the total RNA isolation, the EDTA whole blood samples were centrifuged for 10 min at 5000× *g* at room temperature. The pellet was resuspended in RNAprotect^®^ bacteria reagent (RNeasy^®^ Protect Bacteria Mini Kit, Qiagen, Hilden, Germany) and stored at −80 °C until RNA isolation. The RNA was extracted using the RNeasy^®^ Mini Kit (Qiagen) on a QIAcube extraction robotic system (Qiagen). The quality and quantity of the RNA was assessed using Invitrogen Qubit 3.0 and Nanodrop 2000 (Thermo Fisher Scientific, Waltham, MA, USA).

### 2.2. Biosensor Preparation

We used microcantilever arrays of eight silicon cantilevers (500 μm long, 100 μm wide and 600 nm thick) obtained from IBM Research (Rüschlikon, Switzerland). A previously described procedure [18] for functionalization of cantilever arrays with a receptor layer was applied. Briefly, a piranha solution (30% H_2_O_2_:96% H_2_SO_4_ = 1:1, *v*/*v*) was used to clean arrays for 15 min., rinsed three times with water followed by isopropanol and dried in air. Afterwards the arrays were coated with a 2 nm layer of titanium and 20 nm gold. The gold provides a surface for thiolated oligonucleotides to bind covalently, thus forming a receptor monolayer for highly specific detection of target molecules.

Table 1 shows the oligonucleotides used in all experiments for detection of bacteria in general with 16S-rRNA oligonucleotides and species-specific oligonucleotides for *S. aureus* and *E. coli*, respectively. We used thiol-modified oligonucleotide probes from Microsynth AG (Balgach, Switzerland) at a concentration of 100 µM without Dithiothreitol (DTT). Prior to functionalization oligonucleotides were diluted to a concentration of 20 µM in high-performance liquid chromatography (HPLC) grade DEPC (0.1% diethylpyrocarbonate) treated water (BioConcept, Allschwil, Switzerland) containing 50 mM tri-ethyl-ammonium-acetate (TEAA, obtained from Sigma-Aldrich, St. Louis, MO, USA) buffer and 1 mM tris (2-carboxyethyl)phosphine (TCEP, obtained from Sigma-Aldrich). TCEP was used to reduce disulphide bonds between thiolated oligonucleotide probes. The thiolated oligonucleotides were applied to the cantilevers using a Microdrop inkjet printer (MDP705L, Microdrop Technologies, Norderstedt, Germany) [19]. We used 20 nucleotide long probes in our experiments to ensure better hybridization accessibility, since probes shorter than 24 nucleotides stand upright on the surface forming a self-assembled monolayer (SAM) pinned down covalently by thiol groups [20]. Furthermore, we confirmed full thiol oligonucleotide coverage of the surface using a special etching method [21]. We used a standardized bacteria negative blood reference consisting of 15 individual negative blood samples for establishing the initial baseline before actual patient sample injection.

Upon dispensing and subsequent incubation for an hour at room temperature, the array was mounted in the measurement chamber containing 0.03 × SSC (saline sodium citrate buffer, prepared using 20 × SSC from Sigma Aldrich). Cantilevers can be functionalized individually as required (typically with target specific probes and reference probes). It is of vital importance that differential measurements are performed i.e., the difference of the deflection signals of the probe and reference cantilevers is calculated. External factors such as non-specific interactions and thermal drift are only eliminated by determining differential responses of probe and reference sensors (in this study derivatized with a non-specific polyAC oligonucleotide of the same length as the probe sequence).

**Table 1:** Oligonucleotide probes used in the study.

Probe	Sequence	Use
16S-rRNA	5′-GGACTACCAGGGTATCTAAT-3‘	Bacteria specific detection of 16S-rRNA [22].
polyAC	5′-ACACACACACACACACACAC-3′	Reference sequence for non-specific binding [23].
*femA*	5′-ATAGTGGCCAACAGTTTGCG-3‘	*S. aureus* specific gene sequence [24].
*uidA*	5′-GTAATCACCATTCCCGGCGG-3′	*E. coli* specific gene sequence [25].

### 2.3. Experimental Setup

**Figure 1:**
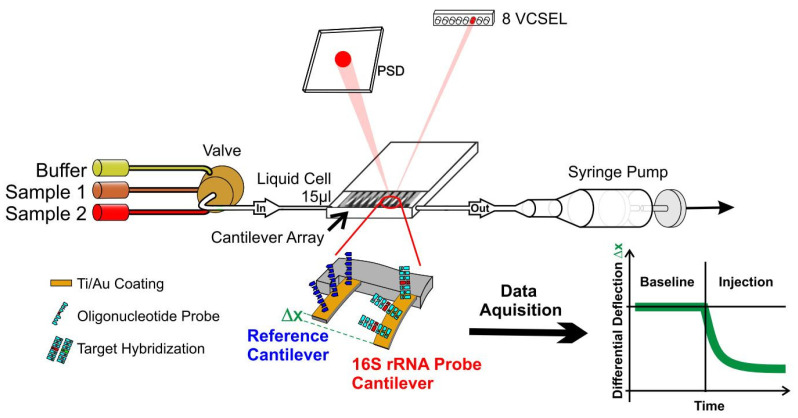
Schematic of a nanosensor measurement. An array of eight gold coated identical silicon cantilevers with 250 µm pitch, a length of 500 µm and a thickness of 600 nm is functionalized with a reference and a 16S-rRNA probe for detection of bacteria. The array is then mounted in a microfluidic cell and exposed to total RNA extracted from a patients’ blood. Bending of probe cantilevers due to hybridization is measured via multiplexed optical beam deflection readout with an accuracy of 0.1 nm. See supplementary material for further pictures and a video.

The setup (Figure 1) consists of a liquid cell containing the sensor array and a multiway valve selector (Rheodyne, Rohnert Park, CA, USA) for buffer and samples. Liquid exchange is controlled by pulling via a syringe pump (GENIE, Kent Scientific, Torrington, CT, USA), allowing different concentrations between 1 fg µL^−1^ and 40 ng µL^−1^ of total RNA to flow at a rate of 20 µL min^−1^ for a total of 200 to 400 µL. All measurements were performed at 27 °C in a temperature-controlled box with a steady buffer flow of 20 µL/min, which was found optimal for this type of experiment. Data acquisition hardware, temperature regulation and a syringe pump for buffer and sample injection were controlled by dedicated LabView software.

Sensor readout is performed by time-multiplexed vertical-cavity surface-emitting lasers (VCSEL) with regulated power supply (operated at 1 Hz, wavelength 760 nm, Avalon Photonics, Zurich, Switzerland) in combination with adjustable optics to yield a light beam pitch of 250 µm adapted to the cantilever pitch. A 2D linear position-sensitive detector, PSD (SiTek Electro Optics, Partille, Sweden) is used to detect the position of the deflected beam. The signals were preamplified, and the data were acquired using a National Instruments (Austin, TX, USA) PCMCIA 16XE-50 (16 bit, 200 kS s^−1^) data-acquisition card. Data processing is done using Origin Software.

### 2.4. Experimental Procedure

Total RNA was dissolved in HPLC grade DEPC (0.1% diethylpyrocarbonate) treated water was further serially diluted in 0.03 × SSC for the experiments. All measurements were performed with the home-built sensor instrument described above [18]. The bending of cantilevers was detected by reflection of an external laser beam focused at the cantilever apex. The instrument enables monitoring the deflection of all eight sensors in parallel in a time-multiplexed manner with sub-nanometer accuracy.

## 3. Results and Discussion

### 3.1. Determination of Level of Rejection

First, we focused on the development of a robust assay. We determined the rejection level and the lower limit of detection for bacteria in EDTA whole blood. For that purpose, we tested blood samples without bacteria and samples containing various concentrations of bacteria.

**Figure 2:**
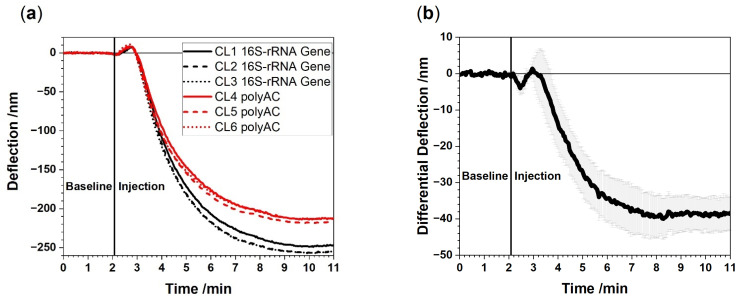
Raw data (absolute deflection) from a representative experiment in which 100 pg/µL of total RNA extracted from EDTA whole blood is injected (**a**). Bending curves of the 16S-rRNA functionalized cantilevers (CL1, CL2, CL3) and the polyAC reference cantilevers (CL4, CL5, CL6). (**b**) Average differential 16S-rRNA signal with corresponding standard deviation obtained from differences in responses of 16S-rRNA vs. polyAC cantilevers. A negative signal evolves due to compressive stress on the surface as the 16S-rRNA functionalized cantilevers caused by specific binding to the probes on the cantilevers. Only through differential deflection measurements the full potential of the method is revealed exhibiting nanometer signals down to 5 nm sensitivity. The signal to noise ratio is about 8:1.

Figure 2 shows a representative cantilever response from sensors exposed to total RNA extracted from a patient with bacteremia. It is of paramount importance to include reference probes to avoid undesired effects such as refractive index changes or nonspecific binding. The differential measurement corrects unwanted artifacts and thereby unambiguously reveals the presence of bacterial 16S-rRNA.

**Figure 3:**
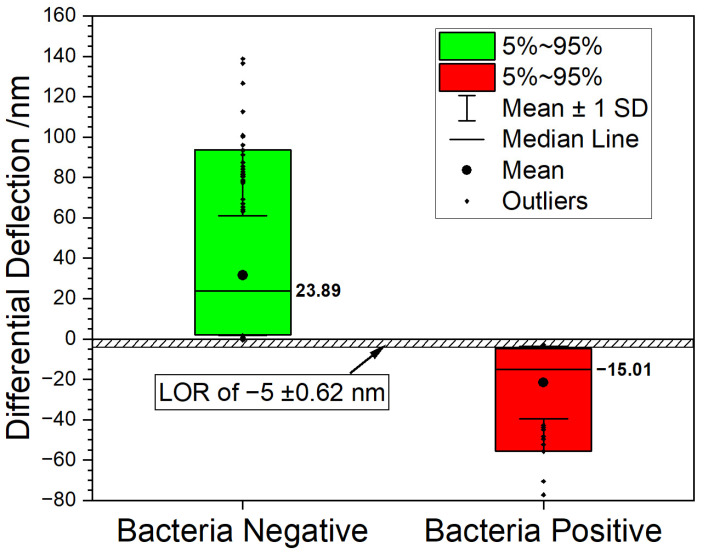
Boxplot graph summarizing the measurements. 90% of all datapoints fall inside the box. The bacteria negative samples (green box) shows a median value of 23.89 nm vs. a median value of −15.01 nm for the bacteria positive samples (red box). The graph shows that the two populations can be clearly distinguished, suggesting a rejection level above −5 nm with a signal to noise ratio of 8:1.

The level of rejection (LOR) is determined by analyzing cantilever responses from negative controls (total RNA extracted from bacteria-free blood samples). The LOR value reflects nonspecific binding of total RNA from human blood to 16S-rRNA specific cantilevers. For this purpose, human blood samples were compared to blood samples containing bacteria (Figure 3). We observe two clearly distinguishable distributions consisting of samples with bacteria (red bars) and without bacteria (green bars), respectively. Negative deflection is considered bacteria positive. Positive deflection values indicate bacteria negative samples. Hence, we can clearly and instantly identify samples with bacteria only using the sign of the deflection value.

### 3.2. Determination of Limit of Detection

**Figure 4:**
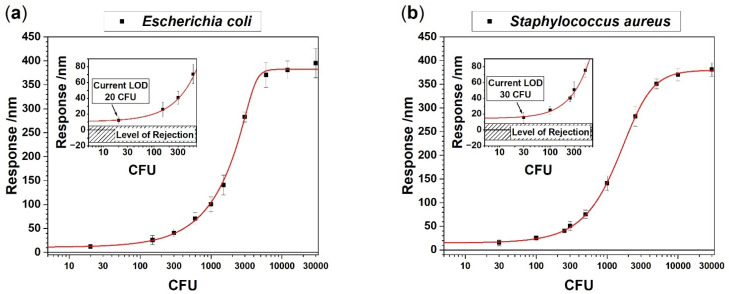
Evaluation of current limit of detection (LOD) in colony forming units (CFU) as measured for total RNA extracted from EDTA whole blood spiked with *E. coli* (**a**) *or S. aureus* (**b**). Each point is the average of 3 measurements. The insets show the LOD measured for *E. coli* as 20 CFU and for *S. aureus* as 30 CFU, respectively. The fitted curves using a logistic Equation (1) indicate a lower LOD of less than 5 CFU. Below that level the LOR will prevent proper analysis.

Based on these observations we determined the technical limit of detection (LOD) using blood samples spiked with a defined number of bacteria (Figure 4) setting predefined concentration values. Since patients with bacteremia may exhibit only a few dozen cells per mL, high sensitivity for detection of bacteria is essential. A logistic Equation (1) was used to evaluate the technical LOD for total RNA extracted from blood samples spiked with different numbers of colony forming units (CFU) of *E. coli* or *S. aureus*. RNA concentrations were adjusted to 100 pg/µL. As each spiked blood sample had a different total RNA concentration after extraction, the number of bacteria had to be calculated accordingly, which explains the unequal distribution of CFU numbers in the *X*-axis values. Please note that the deflection at equilibrium is the same for both species which might indicate a similar amount of 16S-rRNA in each species. Despite the fact that *E. coli* is Gram-negative and *S. aureus* is a Gram-positive species and have different mechanisms for 16S-rRNA expression control [26], we found equal amounts of 16S-rRNA.

(1) $$D\left(c\right)=D_{min}+\frac{D_{max}-D_{min}}{{\left(1+{\left(\frac{c_{0}}{c}\right)}^{h}\right)}^{s}}$$

where *D(c)* is equal to the response in nm at a particular concentration *c*, *D_min_* corresponds to the minimum response and *D_max_* to the maximum response. h corresponds to the Hill coefficient at *c_0_* equal to half *D_max_*, *s* is a control factor.

### 3.3. Characterization of Selected Patient Samples

**Figure 5:**
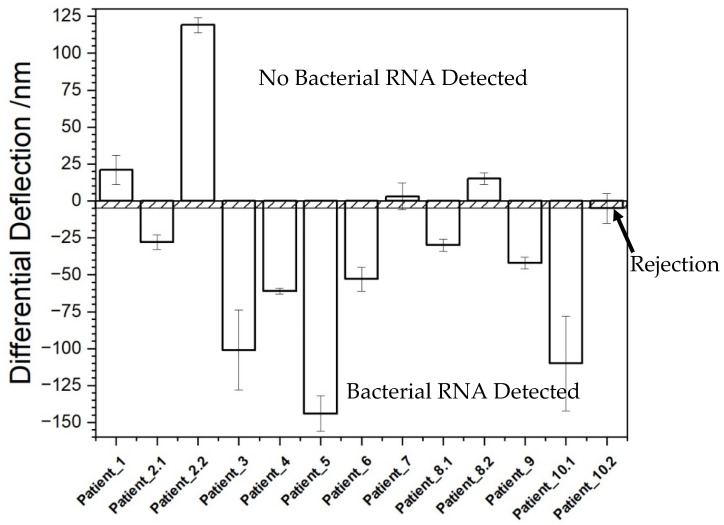
Cantilever responses to different patient samples. These signals allowed to decide on the status of the patients. In all cases the patients’ status was determined correctly with our method.

In the following experiments we examined in detail bacteremia in 13 blood samples from 10 different patients before antibiotic and after antibiotic treatment for bacterial infections (Table 2, Figure 5).

Figure 5 shows the deflection signals of the samples. For patient 2, 8 and 10, 2 samples were collected at different times, one before antibiotic treatment and a second sample a couple of days after the antibiotic treatment was started. The negative signal in the second sample correlates with the blood culture result of the patient which were negative at that point and indicates the successful antibiotic treatment. In all cases the patients’ status was determined correctly with our nanosensor method.

### 3.4. Identification of Different Bacterial Species

**Figure 6:**
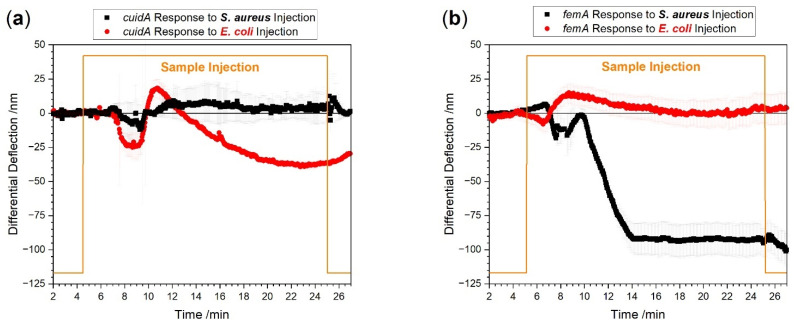
Measurements with species-specific probes directly from EDTA whole blood. Total RNA concentration was adjusted to 100 pg/µL sample. The red curve shows *E. coli* specific responses. *S. aureus* specific responses are shown in black. In panel (**a**), total RNA from an *E. coli* positive patient was used showing a *uidA* specific (red) and no *S. aureus uidA* response (black). In panel (**b**), total RNA from an *S. aureus* positive patient was tested showing an *S. aureus femA* specific (black) and no *E. coli uidA* specific response (red).

Another pivotal aspect is the direct identification of the bacterial species that causes sepsis. For that purpose, we studied two of the most common bacterial species, i.e., *E. coli* and *S. aureus* [27]. For identification of *E. coli*, we used the *uidA* gene which encodes the 1-glucuronidase enzyme [28] which hydrolyses glycosidic bonds in carbohydrates. The *femA* gene is used to identify *S. aureus*. *FemA* encodes an enzyme of the citric acid cycle [29]. Figure 6 shows the measurements with different total RNA samples from patients with *E. coli* and with *S. aureus* infections.

We observe distinctive signals for *E. coli* (Figure 6a) as well as for *S. aureus* (Figure 6b) infected blood samples, respectively. We found a difference in the largest responses to the injection after 20 min between the *E. coli* signal of −37 ± 3 nm and the *S. aureus* signal of −91 ± 10 nm. The response time of *uidA* to reach the largest value is about 8 min whereas the response time to *femA* is only 4 min. The difference in response times and signal magnitudes are indicative of uneven bacterial load between the patient samples. As estimated from the plots in Figure 6a,b, the *E. coli* positive patient has about 250 CFU per milliliter blood and the *S. aureus* positive patient has approximately 600 CFU per milliliter blood. Please note that the limit of detection is much lower, i.e., 20 to 30 CFU for the bacterial species investigated. These numbers are in agreement with published PCR–Electrospray Ionization Mass Spectrometry data [30].

## 4. Conclusions

The work presented showcases the high sensitivity of nanomechanical sensors for detection of bacteremia directly from EDTA whole blood samples from patients without the need for bacterial blood cultures. Our method offers a technical limit of detection down to the equivalent of 20 CFUs/mL at fast response times within less than 10 min in Gram-positive and Gram-negative bacteria. These findings show the wide applicability of nanomechanical biosensors for RNA detection in clinical microbiology and provide substantial faster results than measuring bacterial vibrational motion or other currently applied techniques. We succeeded to identify bacterial species via 16S-rRNA probes as well as the species that caused bacteremia. At a later stage, this technology will be explored for a broader range of bacterial species and antibiotic resistance genes directly in septic patients using a clinical trial.

## Figures and Tables

**Table 2:** Concentrations of total RNA extracted from EDTA whole blood of 10 different patients and determined bacteremia state. Human EDTA whole blood without bacteremia is used as a control. The various colours correspond with the colours in Figure 5. (+) indicates patient samples with bacterial infection before treatment and (−) after treatment diagnosed free of bacterial infection as determined in blood culture based diagnostics (reference standard). N.D.: not detected.

Sample	RNA Concentration (ng/µL)	Strain Detected in Blood Culture	Results from Blood Cultures	Results from Nanosensor Assay
EDTA whole blood (w/o infection, negative control)	1.8	−		−
Patient 1	5.8	*N.D.*	−	−
Patient 2.1	22.8	*E. coli*	+	+
Patient 2.2 (2 days treated)	8.9	*N.D.*	−	−
Patient 3	4.9	*S. aureus*	+	+
Patient 4	1.7	*S. aureus*	+	+
Patient 5	3.1	*E. coli*	+	+
Patient 6	8.6	*E. coli*	+	+
Patient 7	2.6	*N.D.*	−	−
Patient 8.1	11	*S. aureus*	+	+
Patient 8.2 (2 days treated)	10.1	*N.D.*	−	−
Patient 9	17.8	*E. coli*	+	+
Patient 10.1	1.6	*S. aureus*	+	+
Patient 10.2 (1 day treated)	2.5	*N.D.*	−	within LOR

## Data Availability

The data presented in this study are available on reasonable request from the corresponding author.

## Supplementary Materials

The following supporting information can be downloaded at: https://www.mdpi.com/article/10.3390/bios12110994/s1, Figure S1: Nanosensor arrays; Figure S2: Nanosensor liquid cell; Figure S3: Position Sensitive Detector; Figure S4: Nanosensors array setup; Video S1: Rapid bacteria detection from patients’ blood bypassing classical bacterial culturing.

## References

[1] Rudd K.E., Johnson S.C., Agesa K.M., Shackelford K.A., Tsoi D., Kievlan D.R., Colombara D.V., Ikuta K.S., Kissoon N., Finfer S. (2020). Global, regional, and national sepsis incidence and mortality, 1990–2017: Analysis for the Global Burden of Disease Study. Lancet.

[2] Chun K., Syndergaard C., Damas C., Trubey R., Mukindaraj A., Qian S., Jin X., Breslow S., Niemz A. (2015). Sepsis Pathogen Identification. J. Lab. Autom..

[3] Cheng M.P., Stenstrom R., Paquette K., Stabler S.N., Akhter M., Davidson A.C., Gavric M., Lawandi A., Jinah R., Saeed Z. (2019). Blood Culture Results Before and After Antimicrobial Administration in Patients with Severe Manifestations of Sepsis a Diagnostic Study. Ann. Intern Med..

[4] Hinić V., Ziegler J., Straub C., Goldenberger D., Frei R.J. (2015). Extended-spectrum beta-lactamase (ESBL) detection directly from urine samples with the rapid isothermal amplification-based eazyplex (R) SuperBug CRE assay: Proof of concept. Microbiol. Methods.

[5] Nguyen T., Ngo T.A., Bang D.D., Wolff A. (2019). Optimising the supercritical angle fluorescence structures in polymer microfluidic biochips for highly sensitive pathogen detection: A case study on Escherichia coli. Lab Chip.

[6] Murray P.R., Masur H. (2012). Current Approaches to the Diagnosis of Bacterial and Fungal Bloodstream Infections for the ICU. Crit. Care. Med..

[7] Weis C., Cuénod A., Rieck B., Dubuis O., Graf S., Lang C., Oberle M., Brackmann M., Søgaard K.K., Osthoff M. (2022). Direct antimicrobial resistance prediction from clinical MALDI-TOF mass spectra using machine learning. Nat. Med..

[8] Colman R.E., Mace A., Seifert M., Hetzel J., Mshaiel H., Suresh A., Lemmer D., Engelthaler D.M., Catanzaro D.G., Young A.G. (2019). Whole-genome and targeted sequencing of drug-resistant Mycobacterium tuberculosis on the iSeq100 and MiSeq: A performance, ease-of-use, and cost evaluation. PLoS Med..

[9] Lenz T.L., Becker S. (2008). Simple approach to reduce PCR artefact formation leads to reliable genotyping of MHC and other highly polymorphic loci—Implications for evolutionary analysis. Gene.

[10] Opota O., Jaton K., Greub G. (2015). Microbial diagnosis of bloodstream infection: Towards molecular diagnosis directly from blood. Clin. Microbiol. Infect..

[11] Dincer C., Bruch R., Kling A., Dittrich P.S., Urban G.A. (2017). Multiplexed Point-of-Care Testing—xPOCT. Trends. Biotechnol..

[12] Longo G., Alonso-Sarduy L., Rio L.M., Bizzini A., Trampuz A., Notz J., Dietler G., Kasas S. (2013). Rapid detection of bacterial resistance to antibiotics using AFM cantilevers as nanomechanical sensors. Nat. Nanotechnol..

[13] Villalba M.I., Stupar P., Chomicki W., Bertacchi M., Dietler G., Arnal L., Vela M.E., Yantorno O., Kasas S. (2018). Nanomotion Detection Method for Testing Antibiotic Resistance and Susceptibility of Slow-Growing Bacteria. Small.

[14] Huber F., Lang H.P., Backmann N., Rimoldi D., Gerber C. (2013). Direct detection of a BRAF mutation in total RNA from melanoma cells using cantilever arrays. Nat. Nanotechnol..

[15] Zhang J.Y., Lang H.P., Yoshikawa G., Gerber C. (2012). Optimization of DNA Hybridization Efficiency by pH-Driven Nanomechanical Bending. Langmuir.

[16] Huber F., Lang H.P., Glatz K., Rimoldi D., Meyer E., Gerber C. (2016). Fast Diagnostics of BRAF Mutations in Biopsies from Malignant Melanoma. Nano Lett..

[17] Huber F., Lang H.P., Lang D., Wüthrich D., Hinić V., Gerber C., Egli A., Meyer E. (2021). Rapid and Ultrasensitive Detection of Mutations and Genes Relevant to Antimicrobial Resistance in Bacteria. Glob. Chall..

[18] McKendry R., Zhang J., Arntz Y., Strunz T., Hegner M., Lang H.P., Baller M.K., Certa U., Meyer E., Güntherodt H.-J. (2002). Multiple label-free biodetection and quantitative DNA-binding assays on a nanomechanical cantilever array. Proc. Natl. Acad. Sci. USA.

[19] Braun T., Ghatkesar M.K., Backmann N., Grange W., Boulanger P., Letellier L., Lang H.-P., Bietsch A., Gerber C., Hegner M. (2009). Quantitative time-resolved measurement of membrane protein–ligand interactions using microcantilever array sensors. Nat. Nanotechnol..

[20] Steel A., Levicky R., Herne T., Tarlov M. (2000). Immobilization of Nucleic Acids at Solid Surfaces: Effect of Oligonucleotide Length on Layer Assembly. Biophys. J..

[21] Bietsch A., Zhang J., Hegner M., Lang H.P., Gerber C. (2004). Rapid functionalization of cantilever array sensors by inkjet printing. Nanotechnology.

[22] Liu W., Li L., Khan M.A., Zhu F. (2012). Popular molecular markers in bacteria. Mol. Genet. Microbiol. Virol..

[23] Zhang J., Lang H.P., Huber F., Bietsch A., Grange W., Certa U., McKendry R., Güntherodt H.-J., Hegner M., Gerber C. (2006). Rapid and label-free nanomechanical detection of biomarker transcripts in human RNA. Nat. Nanotechnol..

[24] Francois P., Pittet D., Bento M., Pepey B., Vaudaux P., Lew D., Schrenzel J. (2003). Rapid Detection of Methicillin-Resistant Staphylococcus aureus Directly from Sterile or Nonsterile Clinical Samples by a New Molecular Assay. J. Clin. Microbiol..

[25] Schlaman H.R., Risseeuw E., Franke-van Dijk M.E., Hooykaas P.J. (1994). Nucleotide sequence corrections of the uidA open reading frame encoding beta-glucuronidase. Gene.

[26] Kästle B., Geiger T., Gratani F.L., Reisinger R., Goerke C., Borisova M., Mayer C., Wolz C. (2015). rRNA regulation during growth and under stringent conditions inStaphylococcus aureus. Environ. Microbiol..

[27] Tabah A., Koulenti D., Laupland K., Misset B., Valles J., de Carvalho F.B., Paiva J.A., Çakar N., Ma X., Eggimann P. (2012). Characteristics and determinants of outcome of hospital-acquired bloodstream infections in intensive care units: The EUROBACT International Cohort Study. Intensiv. Care Med..

[28] Scalise M.L., Garimano N., Sanz M., Padola N.L., Leonino P., Pereyra A., Casale R., Amaral M.M., Sacerdoti F., Ibarra C. (2022). Detection of Shiga Toxin-Producing Escherichia coli (STEC) in the Endocervix of Asymptomatic Pregnant Women. Can STEC Be a Risk Factor for Adverse Pregnancy Outcomes?. Front. Endocrinol..

[29] Zhang S., Qu X., Tang H., Wang Y., Yang H., Yuan W., Yue B. (2021). Diclofenac Resensitizes Methicillin-Resistant Staphylococcus aureus to β -Lactams and Prevents Implant Infections. Adv. Sci..

[30] Bacconi A., Richmond G.S., Baroldi M.A., Laffler T.G., Blyn L.B., Carolan H.E., Frinder M.R., Toleno D.M., Metzgar D., Gutierrez J.R. (2014). Improved Sensitivity for Molecular Detection of Bacterial and Candida Infections in Blood. J. Clin. Microbiol..

