# Supplementary material for: Rapid Bacteria Detection from Patients’ Blood Bypassing Classical Bacterial Culturing
Source: Biosensors (Basel). 2022 Nov 9;12(11):994. doi: 10.3390/bios12110994 (PMC9688106; doi:10.3390/bios12110994)
Supplement: Supplementary file 1 [file biosensors-12-00994-s001.zip › biosensors-1974642-supplementary-done.pdf]

biosensors

MDPI

Article

Rapid Bacteria Detection from Patients' Blood Bypassing Classical Bacterial Culturing

François Huber [1,*], Hans Peter Lang [1], Stefanie Heller [2], Julia Anna Bielicki [3], Christoph Gerber [1], Ernst Meyer [1] and Adrian Egli [2,4,5]

1 Swiss Nanoscience Institute (SNI), Department of Physics, University of Basel, CH-4056 Basel, Switzerland
2 Applied Microbiology Research (Lab 315), Zentrum für Lehre und Forschung, Department of Biomedicine, University of Basel, CH-4031 Basel, Switzerland
3 University Children's Hospital Basel (UKBB), Department of Medicine, University of Basel, CH-4056 Basel, Switzerland
4 Clinical Bacteriology and Mycology, University Hospital Basel, CH-4031 Basel, Switzerland
5 Institute of Medical Microbiology, University of Zurich, CH-8006 Zurich, Switzerland
* Correspondence: francois.huber@unibas.ch (F.H.)

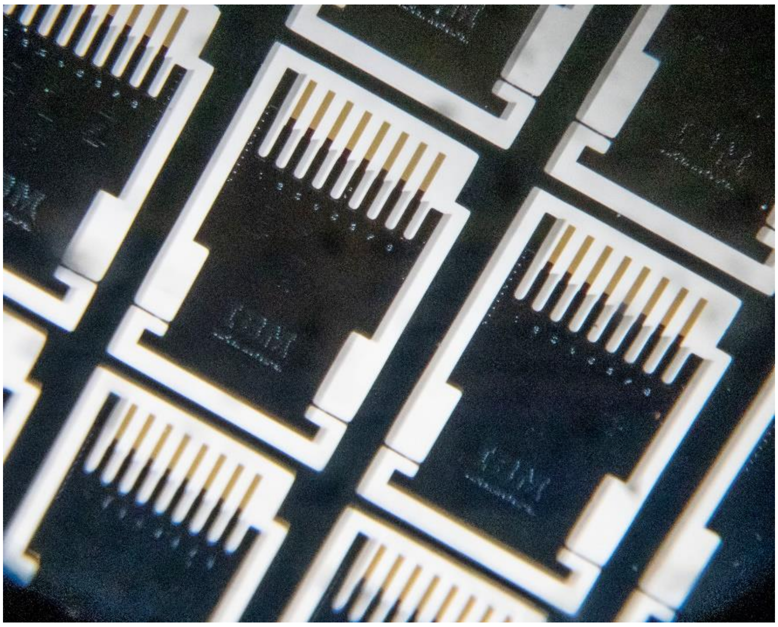

Figure S1. Nanosensor arrays. Arrays with 8 cantilevers, 500 μm long, 600 nm thick and 100 μm wide.

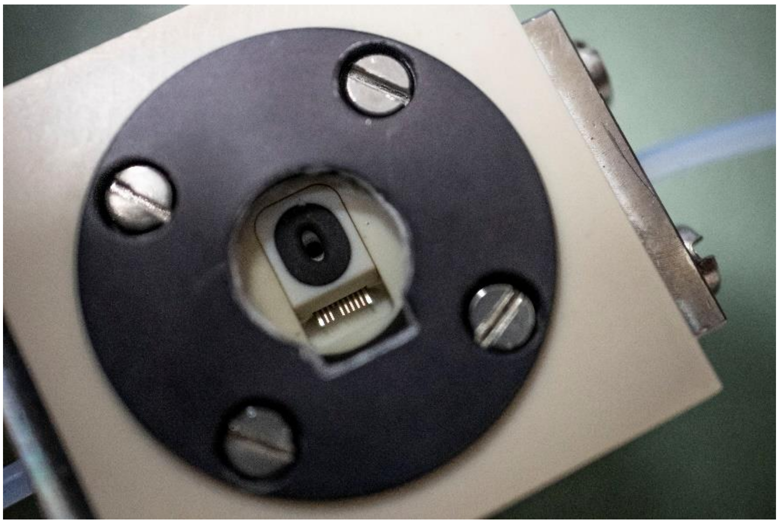

Figure S2. Nanosensor liquid cell with a volume of 15 μL with an inserted nanosensor array.

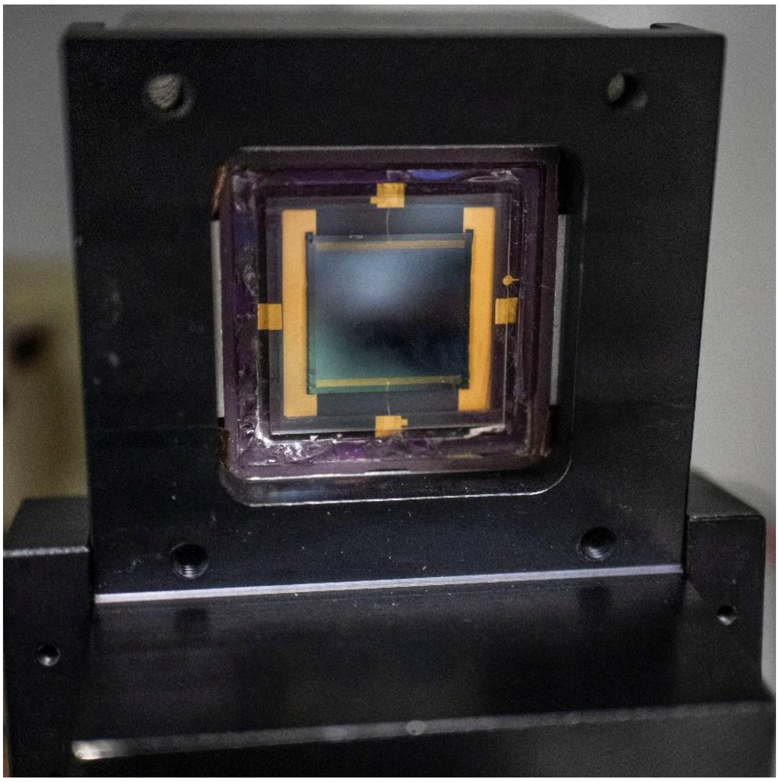

Figure S3. Showing the Position Sensitive Detector (PSD) that measures the cantilever response via laser deflection.

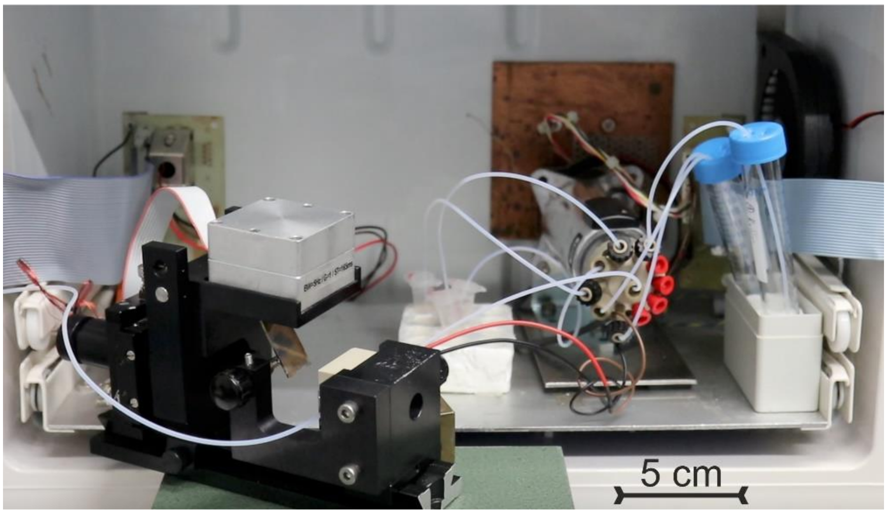

Figure S4. Nanosensors array setup. Showing the liquid cell connected to a multiway valve and liquid samples, the PSD and the VCSEL tube.
